# Motivations behind Gen Z’s news sharing on social media: a PLS-SEM study in Vietnam

**DOI:** 10.3389/fpsyg.2025.1604723

**Published:** 2025-06-18

**Authors:** Le Dinh Hai, Xiao Yan Xiong

**Affiliations:** ^1^School of Journalism and Communication, Hunan Normal University, Changsha, China; ^2^Faculty of Journalism and Communication, Thai Nguyen University of Sciences, Thai Nguyen, Vietnam

**Keywords:** news sharing behavior, Generation Z, fear of missing out (FoMO), news credibility, social media engagement

## Abstract

This study investigates the determinants of news-sharing behavior among Generation Z users on social media in Vietnam by integrating three theoretical frameworks: Newsworthiness Theory, Uses and Gratifications Theory (U&G), and the Theory of Planned Behavior (TPB). A structural equation modeling approach (PLS-SEM) was employed to examine the relationships between perceived news value (social significance, audience relevance), user gratifications (information seeking, socializing, status seeking, entertainment, and pass-time), and psychological drivers such as Fear of Missing Out (FoMO). Data were collected from a stratified random sample of 1.224 high school and university students across six socio-economic regions. The results reveal that social significance, audience relevance, and most gratification-based motivations—excluding pass-time—significantly influence the intention to share news. FoMO was found to positively moderate the impact of information seeking and status seeking on sharing intention. Furthermore, intention to share news significantly predicted actual news-sharing behavior, with inattention to news credibility acting as a mediating factor. The findings underscore the importance of both content attributes and user psychology in shaping digital news sharing among youth. Practical implications are discussed for media organizations aiming to enhance engagement and credibility in the digital era.

## 1 Introduction

In the era of digital transformation, social media has become a central space for news consumption and dissemination (Bhagat and Kim, 2023), particularly among younger generations. Generation Z (Gen Z), born into a digitally connected environment, not only consumes but also actively interacts with news content by sharing, commenting, and curating information across various platforms (Blandi et al., 2022; Devi et al., 2024). In this study, the term news is used broadly to refer to general informational content that spans both hard news (e.g., political, economic, and public policy) and soft news (e.g., entertainment, lifestyle) (Martínez-Costa et al., 2020). This definition aligns with the media consumption habits of Generation Z, who often encounter a mix of news genres on social media platforms (De Los Santos et al., 2025).

As news sharing on social networking sites becomes increasingly prevalent, numerous studies have sought to explain why users share news. Motivations include informing others, expressing opinions, and maintaining social connections (Duffy and Ling, 2020). Additionally, psychological gratifications such as status seeking and social identity play a key role in news engagement (Thompson et al., 2020). Sundar (2008) emphasized that users seek agency and influence, aiming to become opinion leaders in their networks. Kümpel et al. (2015) proposed three categories of motivation: self-serving (e.g., entertainment, self-promotion), altruistic (e.g., information dissemination), and social (e.g., socializing, approval-seeking). Lee and Ma (2012) further identified information seeking, socializing, entertainment, and status seeking as core drivers of news sharing.

The nature of social media platforms significantly affects patterns of news sharing. Private social networks foster discussion-based sharing, while public platforms amplify viral dissemination (Swart et al., 2019). User interactions within localized and interest-based online communities contribute to distinct patterns of news sharing and reception (Swart et al., 2019). Furthermore, Weeks and Holbert (2013) found that engaging with news content—such as following journalists or news organizations—strongly predicts news sharing on social media.

In addition to studies highlighting external motivational factors that encourage people to share news on social media, some research examines the impact of the news content itself by focusing on its intrinsic attributes. Emotional valence and sensationalism are among the key attributes that influence sharing likelihood. Studies suggest that negative news articles are significantly more likely to be shared compared to neutral or positive ones (Watson et al., 2024). Recommender systems and algorithmic curation play a pivotal role in shaping news exposure and engagement, with personalized news feeds increasing user interaction with specific types of content (Johannesson and Knudsen, 2021). Eilders (2006) emphasized that users are drawn to content with high news value, reinforcing the enduring principle that perceived newsworthiness drives dissemination. Consequently, understanding how audiences assess the value of news content is vital to explaining digital news-sharing behavior.

Despite this growing body of research, few studies have integrated content attributes, user motivations, and psychological factors into a single theoretical framework—particularly in non-Western contexts. Moreover, there is a lack of empirical evidence on how Generation Z in developing countries like Vietnam evaluates newsworthiness and responds to psychological factors such as Fear of Missing Out (FoMO). Addressing this gap, the present study investigates the determinants of news-sharing behavior among Generation Z social media users in Vietnam. To develop a comprehensive theoretical framework for understanding Generation Z’s news-sharing behavior on social media, this study conceptually integrates three foundational theories. Newsworthiness Theory (Shoemaker et al., 1991) explains how content attributes—such as social significance and audience relevance—affect perceived value and thus sharing potential. Uses and Gratifications Theory (U&G) contributes a user-centric perspective, focusing on intrinsic motivations such as information seeking, entertainment, and socializing (Park et al., 2009; Lee and Ma, 2012). Meanwhile, the Theory of Planned Behavior (TPB) serves as a behavioral scaffold that links user motivations and perceived news value with actual sharing behavior via intention (Ajzen, 1991). By combining content-centered, motivation-driven, and behavior-oriented lenses, this integrated framework captures the multidimensional nature of news-sharing behavior in the digital environment, especially among Gen Z users whose actions are shaped by both psychological needs and platform dynamics (Stahl and Literat, 2023).

## 2 Literature review and hypotheses

### 2.1 Newsworthiness

The value of news is used as a guide by journalists, who decide which aspects of events are worth reporting on and which aspects are not newsworthy. The value of news has been constantly discussed from the era of print news to the current era of digital news. News value theory provides promising clues to identify which news factors readers might be most interested in. The roots of news value theory can be found in Lippmann (1922), who first described news value as a journalistic property to determine the probability that an item would be news. Later, Galtung and Ruge (1965), Schulz (1982), Shoemaker (1996), and McQuail (2002) re-conceptualized news factors. These news factors vary depending on the authors, but most of them commonly include the event’s proximity (the psychological distance readers feel about events when they are geographically close), social influence (impact of events on the target audience), and unexpectedness (unusual, unpredictable, controversial, or novel events).

Today, news consumers can selectively share news through social media as if they were news curators. As the boundaries between news producers and consumers become blurred in social networking sites, users who are not journalists increasingly share news according to the values created within social networks. According to Boukes et al. (2020), factors such as relevance, timeliness, prominence, proximity, and human interest are often central to discussions on newsworthiness. The way stories are disseminated and consumed online has introduced new factors like viral potential, engagement metrics (e.g., likes, shares, comments), and audience-generated content as key elements in determining newsworthiness (Anderson, 2024). In social networking sites, trust in acquaintances who provide news could be an important factor affecting news shareability (Yang, 2007). Knowing that news value is influenced by social networks, we selected operational user-oriented factors that are easily rendered meaningful by social media users, including social significance and relevance to the audience (Shoemaker, 1996; Harcup and O’Neill, 2001; Park and Lee, 2020).

The first factor that we considered is social significance, which implies that news items have social impact (Shoemaker, 1996). This perspective assumes that the social wave of an event must exceed a certain degree of influence before it is worth reporting (Galtung and Ruge, 1965). News stories with high social significance, emotional appeal, and human-interest elements tend to perform better in terms of user engagement on social platforms (Brown et al., 2020; Park and Kaye, 2021). In this study, we assumed that the more important the news is, the more likely it is to be shared on social media. Second, relevance to the audience is a concept related to meaningful events that can prompt communication among users. This value is cited as the main concept of value for online news, which transcends spatial barriers and is involved in events that are perceived as physically, psychologically, and culturally relevant (Harcup and O’Neill, 2001). Audience behavior and preferences are increasingly shaping what becomes newsworthy. Through interactions on digital platforms, individuals play a role in defining what stories are considered important, leading to a more interactive and dynamic process of news selection (Al-Rawi, 2019). For instance, news outlets may prioritize certain stories based on the demographic or ideological leanings of their audience (Park and Kaye, 2021). In this study, we assume that news that is more relevant to users is more likely to be shared on social media. Thus, the following hypotheses were posited:

*H1*: Social significance has a positive effect on the intention to share news.

*H2*: Audience relevance has a positive effect on the intention to share news.

### 2.2 Gratifications of news sharing

Uses and gratifications theory explains the social and psychological needs of individuals who actively select and use media to gratify their wants (Katz et al., 1973). As emerging new media, along with traditional media, provide users with a wider array of media selection and content, the U&G theory is considered one of the most effective paradigms for identifying motivations underlying media use in mass communication studies (LaRose and Eastin, 2004). This theory assumes that media users are aware of their motivations for selecting various media options and are engaged in media selection. According to this theory, the act of selectively subscribing to news and sharing it with others is associated with active media users. The theory has also been applied in many recent studies of social media and information management (e.g., Lin and Chu, 2021; Kim et al., 2021; Malik et al., 2021).

With time, developments in Internet technologies gave rise to social media. Nov et al. (2010) suggest that out of its many attractive features, social media’s ability to allow individuals to create their own content, converting them from a passive to active audience, stands out as the most worthy one. This has prompted researchers to consider U&G from a social media perspective. Lee and Ma (2012), who studied the relationship between U&G and information sharing, highlight two key points. First, the literature on the relationship between U&G and social media establishes the applicability of the U&G approach in the study of news-sharing behavior. Second, despite media usage reasons varying across individuals, situations, and types of media, motivations for information seeking, socializing, status seeking, and entertainment were explored as gratifications pursuant to news sharing in the social media community of Facebook. In this study, the author considers these four gratifications, plus an additional pass-time gratification that appears particularly well suited to current use of social media (Thompson et al., 2020). In the following sections, we introduce each of these gratifications separately as background to the hypotheses:

#### 2.2.1 Information seeking

Information seeking is the pursuit of information for real-time acquisition and grasp of social trends, which provides users with timely information (Lee and Ma, 2012). The concept of seeking information has changed dramatically with advancements in technology, especially in social media contexts. Information seeking refers to information acquisition, opinions, or suggestions from credible sources such as news, SNS communities, and websites, which provide users with relevant and timely information related to topics (Junaidi, 2020). According to previous studies of uses and gratifications in social media, this motivation was recognized when consumers utilized the Web to obtain training and information (Papacharissi and De Fatima Oliveira, 2012; Whiting and Williams, 2013), similar to self-education and knowledge acquisition (Plume and Slade, 2018). Larger networks tend to be more diverse and link people together for the purpose of information exchange. For instance, social media (e.g., Facebook) is used to circulate information on the COVID-19 pandemic outbreak in some countries (Bento et al., 2020). When engaging in the Internet news environment, information seeking seems to encourage the sharing of helpful information with others. Generation Z information seeking behavior is often driven by social motivations rather than truth-seeking (Hassoun et al., 2023). Thus, we present the following hypothesis:

*H3*: Information seeking has a positive effect on the intention to share news.

#### 2.2.2 Socializing

Socializing refers to the social desire to maintain intimate relationships with others and usually supports social engagement (Baek et al., 2011). Socializing gratification, or social interaction gratification, refers to the desire for connection (Katz et al., 1973). Whiting and Williams (2013) and Lee and Ma (2012) describe social interaction as the need to converse and interrelate with others, which could address the need for belonging. Studies found a positive correlation between news sharing and socializing gratification (Whiting and Williams, 2013), which promotes a sense of connection and community (Malik et al., 2021). Thus, individuals view sharing news as a convenient way to preserve and expand their social networks, as it allows for something to talk about with friends. Gratifications of intimacy and social validation are key motivators for individuals to disclose personal details on online platforms (Lin and Chu, 2021). Choi (2016) states that people’s consumption of news has become a “socially driven activity.” Social isolation and anxiety can increase Gen Z’s willingness to share personal information online (Lyngdoh et al., 2022). Facebook users ask for information or support to maintain weak ties with others via sharing their interests, mutual friends, or relational goals (Jackson, 2020). Taken together, we assume that news found on Facebook is an item which people can socialize about. Thus, we hypothesize that:

*H4*: Socializing has a positive effect on the intention to share news.

#### 2.2.3 Status seeking

Status seeking describes the motivation for people to share information on social media to improve their fame and social reputation. In social media, status is related to feelings of being popular and enhanced self-esteem (Lee and Ma, 2012). Several studies have identified a significant relationship between status seeking behavior and the likelihood of sharing information online. Thompson et al. (2020) found that users, including Gen Z’s who engage in information sharing, are often driven by the perceived social status benefits. Their study concluded that individuals strategically disseminate information to gain recognition, build credibility, and demonstrate expertise within their communities. Yang et al. (2022) examined the role of status seeking during the COVID-19 pandemic, revealing that individuals were more likely to share pandemic-related information as a means of positioning themselves as informed and responsible members of society. Malik et al. (2021) findings indicate that individuals use social media as a tool for self-enhancement and status acquisition. This behavior is influenced by the desire for peer approval, validation, and increased social influence. Therefore, by sharing news information and participating actively in online communication, users may experience improved self-esteem and respect from peers. Hence, we state the following hypothesis:

*H5*: Status seeking has a positive effect on the intention to share news.

#### 2.2.4 Entertainment

Entertainment refers to the use of social media for the purposes of passing time, relieving boredom, relaxing, and being entertained (Park and Lee, 2020). It has been demonstrated to be one of the main factors determining participation on social networking sites in previous studies (Park et al., 2009; Whiting and Williams, 2013). Audiences engage more with news stories that evoke strong emotions or have entertainment value, thereby increasing their likelihood of becoming viral (Al-Rawi, 2019). Reading comments on social media also provides entertainment and information gratification (Husna and Rianto, 2021). Baek et al. (2011) found that individuals shared links on Facebook because they were relaxing and entertaining. Kim (2014) discovered a high correlation between the entertainment gratification and the utilization of a social recommendation tool (the “like” button on Facebook) and suggested that individuals utilize it for joy and expressing their opinions in a happy/positive manner. Such behavior can result in the release of stress and fulfilling the need for entertainment (Lee and Ma, 2012; Wang and Rzeszotarski, 2023). Consistent with the majority of prior work, we hypothesize that:

*H6*: Entertainment has a positive effect on the intention to share news.

#### 2.2.5 Pass-time

Pass-time was set as an important predictor of common social media use (Kircaburun et al., 2018). It is defined as using social media platforms to alleviate boredom and take up time (Whiting and Williams, 2013). Choi (2016), Apuke and Omar (2020) stated that, apart from socialization, pass-time gratification is the most important predictor of news-sharing behavior. Several studies have also indicated that pass-time motivation positively affects personal media engagement (Davis Mersey et al., 2010); users tend to engage in Facebook to satisfy the pass-time motivation (Tsai and Men, 2013). Entertainment and passing time are identified as major gratifications for sharing news, particularly regarding COVID-19 (Bakhtawar, 2022). Based on the aforementioned analysis, the following hypothesis is advanced:

*H7*: Pass-time has a positive effect on the intention to share news.

#### 2.2.6 Fear of missing out

The rise of social media and the Internet in the 21st century has exposed individuals to a wide array of opportunities that make them feel that every act counts and not one opportunity should ever be missed, known as Fear of Missing Out (FoMO), a form of social anxiety caused by the concern of not being in touch with the events in the cyber world (Reyes et al., 2018; Talwar et al., 2019). In the social media context, FoMO is the anxiety about losing the opportunity to participate in social interactions and acquire meaningful experiences (Alt and Boniel-Nissim, 2018); thus, it motivates people to perform actions to maintain or enhance their social status (Zhang et al., 2020). They tend to be afraid of missing out on events, news, and status updates in social networks and tend to urgently keep an eye on them (Abel et al., 2016), the fear of falling behind (Good and Hyman, 2020). A line of studies has supported the positive relationship between FoMO and information-seeking behavior (Albertson and Gadarian, 2015; Wu-Ouyang and Hu, 2022), suggesting that individuals may intentionally seek news to fulfill their need for information. Consistent with the majority of prior work, we hypothesize that:

*H8*: Fear of Missing Out (FoMO) positively moderates the relationship between information seeking and intention to share news.

*H9*: Fear of Missing Out (FoMO) positively moderates the relationship between status seeking and intention to share news.

#### 2.2.7 Intention to share and sharing behavior

The theory of planned behavior (TPB) forecasts behavior by attempting to predict behavioral intention. Realizing that it is difficult to predict actual behavior, Fishbein and Ajzen (1975) instead sought to predict behavioral intention (Ajzen, 1991)—what a person plans to do—as it is a strong predictor of actual behavior (Ho et al., 2015). Here, we extend the model in the context of news sharing. Due to its flexibility in diverse studies of behavioral intentions, the model has been used to examine intention to share and goals of news sharing (Noland, 2020). The two main components of the model are used in this study, and we therefore present the following hypothesis:

*H10*: Intention to share news has a positive effect on news-sharing behavior.

#### 2.2.8 Inattention to news credibility

Inattention to news credibility refers to the degree to which news consumers are indifferent to the reliability of the news. Individuals typically display limited rationality and must decide how much they care about various pieces of information, because it is impossible to process and identify information about all potential alternatives when making decisions. Therefore, humans are naturally indifferent to much of the information available to them (Caplin and Dean, 2015). In the case of news sharing in social media, inattention could refer to indifferent feelings about news sources, authenticity, and the need for information verification. Pennycook et al. (2021) indicates that people do care about accuracy but may fail to consider it when sharing news on social media due to attentional constraints. By consuming news from various channels online, trust in acquaintances who provide news may replace trust in news itself because users have become rationally inattentive to its credibility (Park and Lee, 2020). Hence, we present the following hypothesis:

*H11*: Inattention to news credibility is a positive mediator in the relationship between intention to share news and news-sharing behavior.

**Figure 1:**
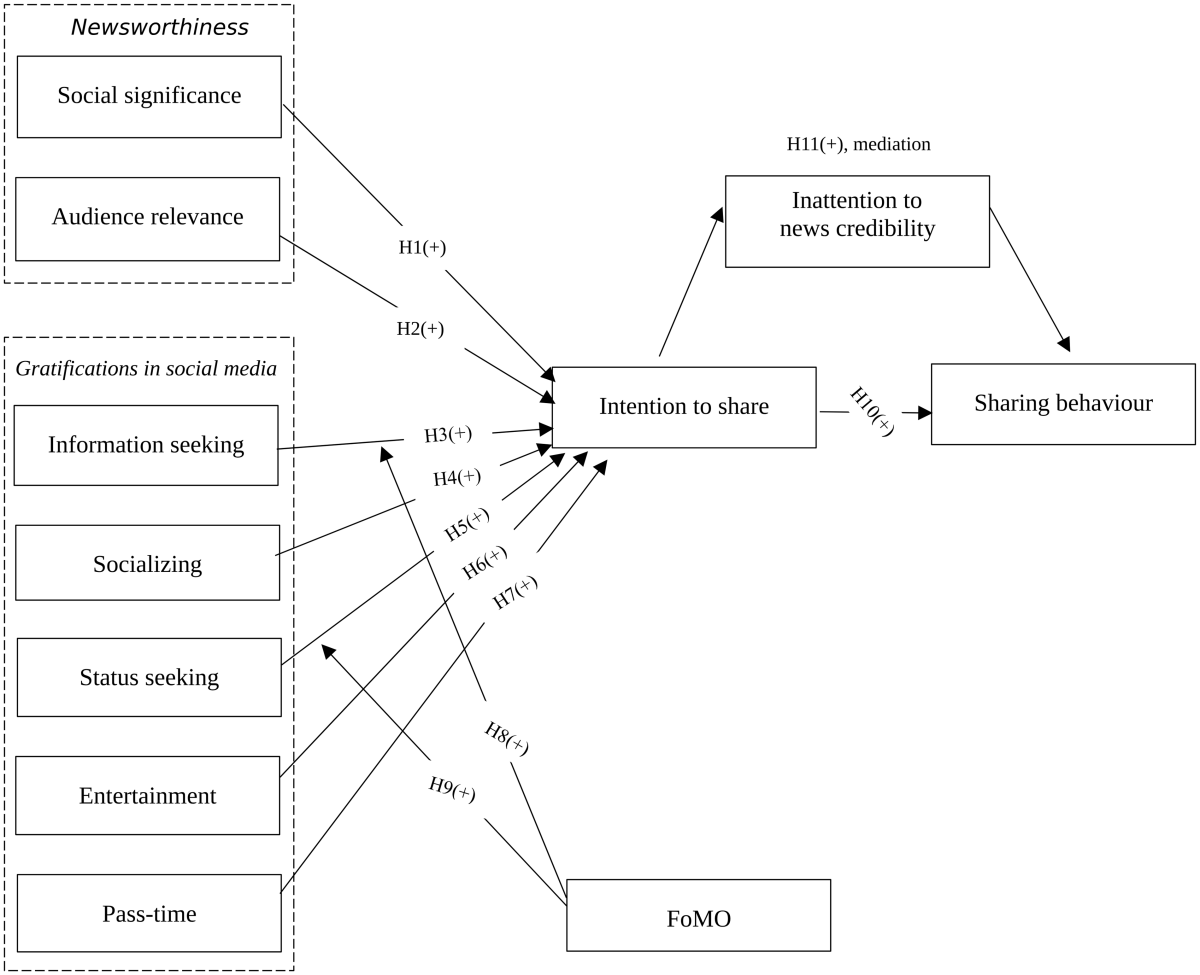
Proposed research model.

We developed a research model as shown in Figure 1, based on our hypotheses.

## 3 Research methodology

The study employed a stratified random sampling method, using geographical regions and socio-economic divisions as the basis for respondent selection. Participants were recruited through a network of collaborators. The research team selected two collaborators from each of the six surveyed cities, resulting in a total of 12 collaborators. After receiving training in survey methodology and data quality standards, the collaborators were responsible for recruiting high school and university students within their respective survey areas. Recruitment at each site ceased once the predetermined sample size was reached. Participants were asked about their sharing behavior regarding a broad spectrum of news content typically encountered on social media, without genre restriction. This approach captures a holistic view of Gen Z’s news sharing.

According to the 2019 Population and Housing Census conducted by the General Statistics Office of Vietnam, the country is divided into six socio-economic regions. Due to the scope of the study and financial constraints, one representative province or city was selected from each region for data collection (General Statistics Office of Vietnam, 2019). Given that the Generation Z population in each selected province or city exceeds 100,000 individuals, the Yamane formula was applied to calculate the required sample size, ensuring the representativeness and scientific rigor of the study (*n* = 204 * 6, total: 1.224); see Table 1 (Yamane, 1969).

**Table 1:** Data collection and demographic analysis.

Constructs	Frequency	Percent
Gender		
Male	516	42.2
Female	708	57.8
Total	1,224	100.0
Age		
15–17	506	41.3
18–21	718	58.7
Total	1,224	100.0
Level of education		
High School Students	506	41.3
University students	718	58.7
Total	1,224	100.0

Their ages ranged from 15 to 21 years (*M* = 18.42, SD = 2.07), with 42.2% identifying as male and 57.8% as female. Of the total sample, 41.3% were aged between 15 and 17, while 58.7% were aged between 18 and 21. As shown in Table 1, a survey instrument was used to accomplish the research goals through a thorough analysis. There were 11 constructions and 44 indicators total. Specifically, items measuring social significance (SS) were adapted from Shoemaker (1996), while items measuring audience relevance (AR) were adapted from Harcup and O’Neill (2001). The scales assessing gratifications related to information seeking (IS), socializing (SI), status seeking (SSE), entertainment (SNE), and passing time (PT) in the context of news sharing on social media were adapted from prior Uses and Gratifications (U&G) research (Lee and Ma, 2012; Park and Lee, 2020; Thompson et al., 2020). Fear of missing out – FoMO (FM) was measured using three items adapted from Reyes et al. (2018) and Talwar et al. (2019). Additionally, the scale items for inattention to news credibility (INC) were adopted and modified from Yang (2007) and Park and Lee (2020). Intention to share news (INS) was operationalized using five items derived from studies by Baek et al. (2011), Park and Lee (2020), and Thompson et al. (2020). Finally, items measuring news-sharing behavior (SNB) were adopted from Chow and Chan (2008) and Park and Lee (2020). All items were rated on a five-point Likert scale, ranging from 1 (“strongly disagree”) to 5 (“strongly agree”).

PLS-SEM was selected for this study due to its suitability for exploratory research and its capacity to handle complex models involving multiple latent constructs and relationships (Hair et al., 2021). The analysis was conducted using SmartPLS software (version 3.3.3), employing a variance-based estimation approach, which is particularly appropriate for predictive modeling (Hair et al., 2019). The evaluation process followed the standard two-step procedure in PLS-SEM: (1) assessment of the measurement model and (2) assessment of the structural model (Hair et al., 2019). This comprehensive procedure ensures methodological rigor and supports robust inferences about both direct and indirect relationships among the constructs. The measurement model was validated through checks for the reliability and validity of latent constructs and their indicators. Specifically, the analysis included outer loadings, Cronbach’s alpha (*α*), composite reliability (CR), and average variance extracted (AVE) to assess internal consistency and convergent validity. Discriminant validity was verified using the heterotrait-monotrait (HTMT) ratio as recommended by Henseler et al. (2015). Additionally, variance inflation factor (VIF) values were examined to detect potential multicollinearity among predictor constructs. For the structural model, the model’s explanatory power was assessed through *R*^2^ values and adjusted *R*^2^ values for endogenous constructs, while effect sizes (*f*^2^) were calculated to determine the relative contribution of each exogenous construct. The Standardized Root Mean Square Residual (SRMR) was reported as an approximate measure of model fit (Henseler et al., 2015). Path coefficients, *t*-values, and *p*-values were estimated to evaluate the proposed hypotheses. Statistical significance was determined using a bootstrapping procedure with 5,000 subsamples to ensure the stability of estimates (Yuan, 2012), based on recommended thresholds (t > 1.96; *p* < 0.05) (Falk and Miller, 1992).

## 4 Results and analysis

### 4.1 The measurement model assessment

The values of the measures, CR, AVE, and outer loading that characterize the convergent validity and inner consistency test for the reflective variables are shown in Tables 2, 3. Some variables AR3, INC3, PT1, PT3, SNE2 and SSE3, were removed due to factor loadings below 0.7. We see that the outside loadings are higher than the 0.7-percent minimal limit (Hair et al., 2019). In turn, this validates the indicator’s reliability. Every composite reliability value and the value are significantly higher than the reference value of 0.7 (Hair et al., 2019). This demonstrates the internal consistency of all constructs. All AVE values are higher than the threshold of 0.5 (Henseler et al., 2014), confirming the model’s convergent validity. The interval [0.029, 0.626] encompasses all HTMT values that demonstrate discriminant validity, satisfying the conservative requirement that they must be less than 0.85 (Henseler et al., 2015). This conclusion is reflected in Table 4, which supports the claim that each construct is unique from the others in accordance with the criteria of empirical research (Hair et al., 2019; see Table 4).

**Table 2:** Factor loadings of constructs.

Constructs	AR	FM	FM*IS	FM*SSE	INC	INS	IS	PT	SI	SNB	SNE	SS	SSE
AR1	0.767												
AR2	0.834												
AR4	0.836												
AR5	0.827												
FM1		0.888											
FM2		0.906											
FM3		0.912											
INC1					0.895								
INC2					0.890								
INC4					0.882								
INS1						0.827							
INS2						0.851							
INS3						0.850							
INS4						0.799							
INS5						0.856							
IS * FM			1.235										
IS1							0.857						
IS2							0.872						
IS3							0.869						
IS4							0.840						
PT2								0.892					
PT4								0.881					
PT5								0.886					
SI1									0.822				
SI2									0.718				
SI3									0.846				
SNB1										0.883			
SNB2										0.881			
SNB3										0.908			
SNE1											0.857		
SNE3											0.878		
SNE4											0.879		
SS1												0.867	
SS2												0.863	
SS3												0.881	
SSE * FM				1.420									
SSE1													0.861
SSE2													0.887
SSE4													0.880
SSE5													0.880

The asterisk “*” indicates moderating effects of Fear of Missing Out (FoMO).

**Table 3:** Construct reliability and validity.

Constructs	Cronbach’s Alpha	Composite reliability	Average variance extracted (AVE)
AR	0.833	0.889	0.667
FM	0.885	0.929	0.813
FM*IS	1.000	1.000	1.000
FM*SSE	1.000	1.000	1.000
INC	0.868	0.919	0.791
INS	0.893	0.921	0.700
IS	0.882	0.919	0.739
PT	0.864	0.917	0.786
SI	0.722	0.839	0.635
SNB	0.870	0.920	0.793
SNE	0.842	0.905	0.760
SS	0.840	0.904	0.758
SSE	0.900	0.930	0.769

The asterisk “*” indicates moderating effects of Fear of Missing Out (FoMO).

**Table 4:** Discriminant validity evaluation for the reflective variables by HTMT criterion.

Constructs	AR	FM	FM*IS	FM*SSE	INC	INS	IS	PT	SI	SNB	SNE	SS	SSE
AR													
FM	0.300												
FM*IS	0.317	0.464											
FM*SSE	0.314	0.029	0.466										
INC	0.178	0.523	0.329	0.248									
INS	0.560	0.557	0.462	0.321	0.367								
IS	0.617	0.162	0.185	0.312	0.036	0.507							
PT	0.370	0.626	0.413	0.223	0.513	0.480	0.154						
SI	0.516	0.226	0.325	0.183	0.126	0.472	0.507	0.225					
SNB	0.343	0.460	0.336	0.272	0.425	0.550	0.330	0.512	0.247				
SNE	0.542	0.529	0.377	0.283	0.378	0.573	0.391	0.544	0.354	0.466			
SS	0.617	0.218	0.257	0.227	0.087	0.527	0.615	0.223	0.493	0.259	0.381		
SSE	0.395	0.610	0.356	0.093	0.492	0.590	0.310	0.580	0.317	0.510	0.548	0.390	

The asterisk “*” indicates moderating effects of Fear of Missing Out (FoMO).

### 4.2 The structural model assessment

The VIF scores for all construct combinations are displayed in Table 5. The greatest value, which falls under the conservative upper limit of 3 (Becker et al., 2015), is 1.947. Therefore, no issues with predictor construct collinearity were found. With SRMR value = 0.078 < 0.08, the research model fits the data.

**Table 5:** Collinearity evaluation between the predictor constructs by inner VIF values.

Constructs	INC	INS	SNB
AR		1.815	
FM		1.947	
FM*IS		1.750	
FM*SSE		1.534	
INC			1.117
INS	1.000		1.117
IS		1.740	
PT		1.738	
SI		1.362	
SNE		1.693	
SS		1.638	
SSE		1.783	

The asterisk “*” indicates moderating effects of Fear of Missing Out (FoMO).

According to Falk and Miller (1992), an *R*^2^ value of 0.10 is considered the minimum acceptable threshold for endogenous constructs in behavioral research. As shown in Table 6, both the *R*^2^ and adjusted *R*^2^ values for Inattention to News Credibility (INC), Intention to Share News (INS), and News Sharing Behavior (SNB) exceed this threshold, indicating that the model demonstrates an acceptable level of explanatory power for each dependent construct. To more accurately assess the model’s explanatory capacity, adjusted R^2^ values were reported alongside standard *R*^2^ values. Unlike unadjusted *R*^2^, which may be inflated by the number of predictors, adjusted *R*^2^ accounts for model complexity and thus provides a more conservative and reliable estimate of explained variance—particularly important in structural models involving multiple latent variables (Hair et al., 2019). The adjusted R^2^ values for INC, INS, and SNB were 0.104, 0.522, and 0.286, respectively. These results suggest that the model explains 52.2% of the variance in intention to share news, reflecting a substantial degree of explanatory power for this construct. Meanwhile, 28.6% of the variance in actual news-sharing behavior is accounted for, indicating a moderate level of predictive relevance. Although the adjusted *R*^2^ for INC is comparatively lower (0.104), it still meets the minimum threshold, suggesting that additional unobserved factors may influence attention to news credibility.

**Table 6:** *R*^2^ and *R*^2^ adjusted.

Constructs	*R* square	*R* square adjusted
INC	0.105	0.104
INS	0.526	0.522
SNB	0.288	0.286

The *f*^2^ effect size is used to test the effect sizes of the outcome variables (Table 7). 0.35, 0.15, and 0.02 are acknowledged as having large, medium, and moderate effects, respectively (Hair et al., 2019). Cohen (2013) went on to say that values less than 0.02 have no impact. Table 7 displays the effect size of pathways ranging from no effect to a considerable influence based on these characteristics. The results indicate that while intention to share news (INS) has a medium effect on news-sharing behavior (SNB), most other relationships exhibit small or no effect. The findings suggest that inattention to news credibility (INC) and fear of missing out (FM) play a minor role in shaping news-sharing behavior, whereas intention to share (INS) serves as a more substantial predictor.

**Table 7:** The result of *f*^2^.

Constructs	INC	INS	SNB
AR		0.008	
FM		0.051	
FM*IS		0.009	
FM*SSE		0.016	
INC			0.071
INS	0.117		0.211
IS		0.024	
PT		0.001	
SI		0.012	
SNE		0.010	
SS		0.020	
SSE		0.040	

The asterisk “*” indicates moderating effects of Fear of Missing Out (FoMO).

### 4.3 Testing of research hypotheses

The analysis results indicate that the majority of direct relationships between latent variables in the model are statistically significant, as evidenced by *p*-values less than 0.05. Only the relationship PT → INS has a *p*-value of 0.475 (< 0.05), indicating that it is not statistically significant. In addition to direct effects, the model also includes several indirect pathways through mediating variables. Specifically, the indirect effects of PT → INS → SNB, PT → INS → INC, and PT → INS → INC → SNB are not statistically significant, as their p-values exceed the 0.05 threshold. In contrast, all other indirect effects in the model demonstrate statistical significance, with p-values below 0.05. Moreover, the original sample (O) coefficients for all significant paths are positive, indicating that these relationships exert a positive influence in the structural model (see Table 8).

**Table 8:** Results of hypotheses test.

Hypothesis	*H*	*β*	*T*-values	*p*-values	Decision
Social significance - > Intention to share news	H1	0.126	4.009	0.000	Supported
Audience relevance - > Intention to share news	H2	0.084	2.350	0.019	Supported
Information seeking - > Intention to share news	H3	0.140	4.481	0.000	Supported
Socializing - > Intention to share news	H4	0.088	3.284	0.001	Supported
Status seeking - > Intention to share news	H5	0.183	4.970	0.000	Supported
Entertainment - > Intention to share news	H6	0.090	2.479	0.013	Supported
Pass time - > Intention to share news	H7	0.026	0.714	0.475	Not supported
Fear of Missing Out (FoMO) * Information seeking - > Intention to share news	H8	0.069	2.195	0.028	Supported
Fear of Missing Out (FoMO) * Status seeking - > Intention to share news	H9	0.075	3.017	0.003	Supported
Intention to share news - > News-sharing behavior	H10	0.410	11.334	0.000	Supported
Intention to share news - > Inattention to news credibility - > News-sharing behavior	H11	0.077	5.872	0.000	Supported

The asterisk “*” indicates moderating effects of Fear of Missing Out (FoMO).

## 5 Discussion and conclusion

**Figure 2:**
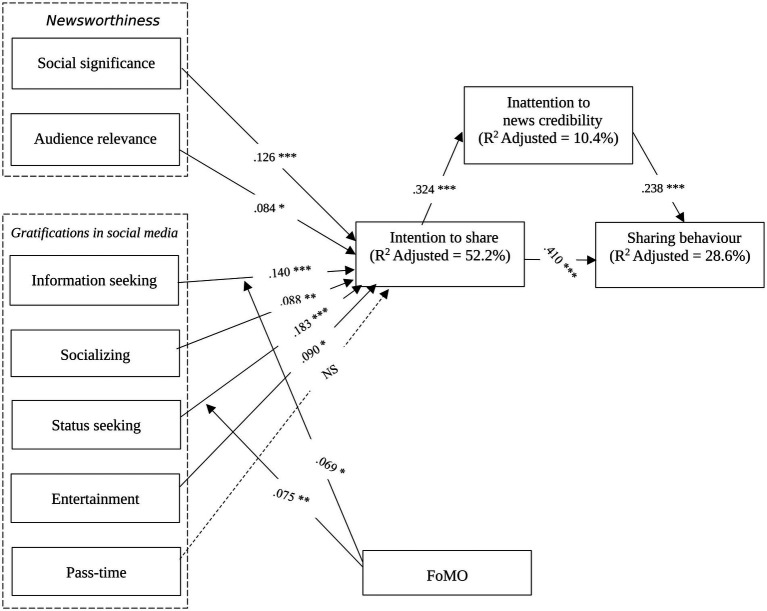
Research model and hypotheses. * *p* < 0.05, ** *p* < 0.01, *** *p* < 0.001.

This study examined the factors influencing Generation Z’s news-sharing behavior on social media in Vietnam by integrating Newsworthiness Theory, Uses and Gratifications Theory (U&G), and the Theory of Planned Behavior (TPB). The findings provide empirical evidence that both news characteristics (social significance and audience relevance) and user motivations (information seeking, socializing, status seeking, entertainment, pass-time, and FoMO) significantly contribute to the intention to share news, which in turn predicts actual news-sharing behavior. Additionally, the study highlights the role of inattention to news credibility (INC) as a mediator, reflecting how credibility perceptions influence digital news sharing (see Figure 2).

In line with Newsworthiness Theory, social significance (H1) and audience relevance (H2) positively influence the intention to share news, consistent with previous studies (Eilders, 2006; Boukes et al., 2020). These findings suggest that news items perceived as socially impactful and personally relevant are more likely to be disseminated by Gen Z’s users. However, compared to earlier research on news-sharing behavior (e.g., Kümpel et al., 2015; Lee and Ma, 2012), this study reveals that newsworthiness factors alone are not the strongest predictors of news-sharing behavior. Instead, intrinsic user motivations (gratifications) play a more substantial role, aligning with contemporary discussions on audience-driven news curation (Swart et al., 2019).

The findings demonstrate that information seeking (H3), socializing (H4), status seeking (H5), and entertainment (H6) positively affect the Generation Z’s in Vietnam intention to share news, reinforcing U&G theory’s application to social media environments (Papacharissi and De Fatima Oliveira, 2012; Duffy and Ling, 2020). Information seeking remains a key motivator, as users actively share news to stay informed and provide others with valuable information (Johannesson and Knudsen, 2021). Socializing plays a significant role, as news sharing fosters online interactions and strengthens social connections (Choi, 2016). Status seeking, while significant, has a smaller effect size than expected, suggesting that self-enhancement motivations may be more context-dependent, influenced by trending news topics and social validation mechanisms (Thompson et al., 2020). Interestingly, the effect of pass-time gratification (H7) on the intention to share news was not statistically significant (*p* = 0.475). This finding diverges from previous research (e.g., Apuke and Omar, 2020; Bakhtawar, 2022), which identified pass-time as a prominent driver of news-sharing behavior on social media. A potential explanation for this discrepancy lies in the behavioral tendencies of Generation Z in Vietnam, who may approach news sharing more purposefully. Rather than engaging in news sharing as a means of alleviating boredom, these users are likely to be driven by more active motivations, such as information seeking, social interaction, status enhancement, or contributing to online communities—indicating a more deliberate and goal-oriented pattern of engagement.

This study contributes novel insights by examining FoMO as a moderator in social media news sharing. The results indicate that FoMO strengthens the relationship between information seeking and intention to share (H8), suggesting that users who fear missing out on trending news are more likely to engage in active information-sharing behaviors (Zhang et al., 2020). FoMO also strengthens the status seeking effect on news sharing intention (H9), aligning with studies that indicate that individuals seek social inclusion through engaging with widely discussed news topics (Alt and Boniel-Nissim, 2018). These findings highlight that FoMO is a unique psychological driver in social media news-sharing behavior, particularly among younger audiences (Generation Z’s) immersed in algorithm-driven, real-time news ecosystems.

Consistent with TPB (Ajzen, 1991), the study confirms that intention strongly predicts actual news-sharing behavior (H10, *β* = 0.410, *p* < 0.001), validating the intention-behavior pathway in social media contexts. However, the lower adjusted R^2^ values for actual behavior (0.286) compared to intention (0.522) suggest that external influences, such as algorithmic curation, peer engagement, etc., may play additional roles in news sharing decisions.

A key contribution of this study is identifying Generation Z’s inattention to news credibility (H11) as a positive mediator between intention to share and actual sharing behavior. Users often prioritize engagement metrics (likes, shares) over source credibility when sharing news (Pennycook et al., 2021), and trust in personal networks can override concerns about information accuracy, leading to the spread of unverified content (Park and Lee, 2020). The result suggests that credibility heuristics in social media news sharing require further investigation, particularly in the context of misinformation and selective exposure biases.

Theoretically, this study advances the literature on digital news-sharing behavior in three key ways. First, it integrates content-level (newsworthiness), user-level (psychological gratifications), and behavioral-level (intention) constructs into a unified model, offering a comprehensive framework for understanding social media–based news sharing. Second, it introduces Fear of Missing Out (FoMO) as a novel moderating variable, demonstrating its role in amplifying the effects of both information seeking and status seeking on sharing intention. Third, it identifies inattention to news credibility as a mediating factor between intention and behavior, shedding light on why users may engage in news dissemination without verifying source reliability. By combining cognitive (Newsworthiness Theory), psychological (Uses and Gratifications Theory), and behavioral (Theory of Planned Behavior) dimensions, the study offers a multidimensional understanding of how Generation Z in Vietnam engages with news on social media.

Practically, the findings carry several important implications for media organizations, digital platform designers, and educators. As social media has become a dominant channel for news distribution, understanding what motivates users to share news is critical for engagement strategies. The results suggest that Generation Z users are more likely to share news to enhance social status rather than purely for interpersonal connection. The finding indicates that content strategies aimed at increasing sharing and virality should cater to status-driven motivations—such as enabling users to associate themselves with timely, insightful, or socially impactful content. To capitalize on news-sharing behavior, media organizations should optimize content for audience relevance and social significance, ensuring that news resonates with Gen Z’s values and aspirations. Moreover, given the observed inattention to credibility, especially under social validation pressures, platforms and media organizations should implement visible credibility indicators—such as fact-check labels, verified sources, or contextual warnings—to help guide more informed sharing decisions. Finally, these insights are also relevant for digital citizenship education. As Gen Z increasingly shapes public discourse through news sharing, equipping them with the skills to critically evaluate content, recognize algorithmic influence, and navigate psychological pressures like FoMO is vital to encouraging responsible and ethical media engagement in digital environments.

## 6 Limitations and future research

Despite the valuable insights provided, this study has several limitations that warrant consideration and offer opportunities for future research.

While the research model provides meaningful insights into Generation Z’s news-sharing behavior, the explained variance for certain constructs remains modest—specifically, the adjusted R^2^ values for Inattention to News Credibility (INC) and News Sharing Behavior (SNB) are 0.104 and 0.286, respectively. Although these values exceed the minimum threshold for behavioral research (Falk and Miller, 1992), they indicate that additional psychological, social, or contextual variables may substantially influence these outcomes. Future studies should consider integrating broader cognitive factors (e.g., trust in institutions, perceived news overload) or environmental variables (e.g., media literacy, platform regulation) to enhance explanatory power.

The effect sizes (*f*^2^ < 0.05) for key intrinsic motivations—such as information seeking, socializing, and status seeking—suggest that while statistically significant, these factors exert relatively limited practical influence on intention. This implies that intrinsic gratifications alone may be insufficient to explain the complexity of Gen Z’s news-sharing behaviors, particularly in algorithmically mediated and socially performative digital environments. Future models could incorporate additional constructs, such as emotional valence, perceived social pressure, or habitual engagement, to capture more nuanced motivational patterns.

This study’s sample was limited to Generation Z social media users in Vietnam, which may restrict the generalizability of findings to other contexts. Cultural factors such as collectivism, peer influence, and high social media penetration likely shape sharing behaviors in distinct ways compared to more individualistic societies. Future research should adopt cross-cultural comparative approaches to explore how these motivations vary across digital and sociopolitical environments.

Additionally, reliance on self-reported survey data introduces potential biases, including social desirability and recall inaccuracy. Although anonymity was maintained to mitigate these effects, future studies may benefit from combining self-report measures with behavioral tracking, digital trace data, or experimental designs to validate findings and reduce common method variance.

The cross-sectional design also limits causal inference. While associations were identified, temporal relationships remain untested. Longitudinal or experimental designs are recommended to investigate how motivations and behaviors evolve over time.

Lastly, the study does not differentiate between platforms, despite evidence that platform affordances influence sharing behavior. For example, TikTok encourages viral, entertainment-focused sharing (Meng, 2021), Facebook supports discussion-based news engagement (Kim, 2024), and Instagram emphasizes status-driven, visual content (Pooja and Ajay, 2025). Future research should examine platform-specific dynamics to better understand and engage Gen Z audiences.

## Data Availability

The original contributions presented in the study are included in the article/Supplementary material, further inquiries can be directed to the corresponding author.
